# SARS-COV-2 detection in saliva and nasopharyngeal swabs using RT-PCR was similar

**DOI:** 10.1590/0103-6440202204591

**Published:** 2022-04-29

**Authors:** Taísa Coelho Guimarães, Barbara Bruno Fagundes Marques, Justine Monteiro Monnerat Tinoco, Luís Cristóvão Moraes Sobrino Porto, Eduardo Muniz Barretto Tinoco, Ricardo Guimarães Fischer

**Affiliations:** 1 Department of Periodontology, Rio de Janeiro State University, Brazil; 2 Histocompatibility and Cryopreservation Laboratory, Rio de Janeiro State University, Brazil; 3 Department of Endodontic, Rio de Janeiro Federal University, Brazil; 4 Clinical Pathology Service, Piquet Carneiro Policlinic, Rio de Janeiro State University, Brazil

**Keywords:** saliva, real-time RT-PCR, nasopharyngeal swab, Covid-19, SARS CoV-2

## Abstract

The World Health Organization has declared the widespread spread of SARS-CoV-2 and its associated disease (COVID-19) a public health emergency. The standard gold test for detecting the virus is the RT-PCR, performed from nasopharyngeal swab (NPS) samples. However, this test may be uncomfortable for the patient and requires specific training and attire from the health professional responsible for collecting the sample. Therefore, the search for alternative ways to collect samples that may be used in the diagnosis of COVID-19 is relevant. This study aimed to compare the results obtained from NPS and saliva samples. NPS and saliva samples were collected from 189 symptomatic outpatients suspected of COVID-19, who came to Piquet Carneiro Polyclinic. RNA extraction was performed using the Bio-Gene DNA/RNA Viral Extraction kit (Bioclin®). Real-time reverse transcriptase-polymerase chain reaction (RT-PCR) reactions used the Molecular SARS-CoV-2 (E / RP) kit (Bio-Manguinhos). The results indicated that 142 showed a non-detectable result (ND), while 47 showed a detectable result (D). Among the 142 "ND", 137 (94.4%) saliva samples obtained the same result, while 5 samples (3.4%) were "D". Among the 47 "D" swab samples, 35 (74.4%) showed the same result in the saliva samples. The sensitivity of the saliva test was 0.74 and the specificity was 0.97. The positive predictive value was 0.88 while the negative predictive value was 0.92. The results showed that detection of Sars-CoV-2 using saliva samples showed high sensitivity and specificity compared to nasopharyngeal swabs.

## Resumo

A Organização Mundial da Saúde declarou a disseminação generalizada do SARS-CoV-2 e sua doença associada (COVID-19) uma emergência de saúde pública. O teste padrão ouro para detecção do vírus é o RT-PCR, realizado a partir de amostras de swab nasofaríngeo (NPS). No entanto, esse exame pode ser desconfortável para o paciente e requer treinamento específico e vestimenta do profissional de saúde responsável pela coleta da amostra. Portanto, a busca por formas alternativas de coleta de amostras que possam ser utilizadas no diagnóstico de COVID-19 é relevante. O objetivo deste estudo foi comparar os resultados obtidos em amostras de NPS e saliva. Amostras de NPS e saliva foram coletadas de 189 pacientes ambulatoriais sintomáticos com suspeita de COVID-19, que procuraram a Policlínica Piquet Carneiro. A extração de RNA foi realizada com o kit Bio-Gene DNA / RNA Viral Extraction (Bioclin®) e as reações em tempo real da reação em cadeia da polimerase-transcriptase reversa (RT-PCR) usaram o kit Molecular SARS-CoV-2 (E / RP) (Bio-Manguinhos). Os resultados indicaram que 142 apresentaram resultado não detectável (ND), enquanto 47 apresentaram resultado detectável (D). Entre os 142 "ND", 137 (94,4%) amostras de saliva obtiveram o mesmo resultado, enquanto 5 amostras (3,4%) foram "D". Dentre as 47 amostras de swab "D", 35 (74,4%) apresentaram o mesmo resultado nas amostras de saliva. A sensibilidade do teste de saliva foi de 0,74 e a especificidade foi de 0,97. O valor preditivo positivo foi de 0,88, enquanto o valor preditivo negativo foi de 0,92. Os resultados mostraram que a detecção de Sars-CoV-2 em amostras de saliva apresentou alta sensibilidade e especificidade quando comparada com swabs nasofaríngeos.

## Introduction

Concerned by the alarming levels of spread and severity, on March 11, 2020, the World Health Organization (WHO) declared the outbreak of COVID-19 a pandemic. The disease has spread worldwide and there have been over 226 million reported cases and 4.6 million deaths on September 16, 2021 ^1^.

Testing individuals for COVID-19 has been an important activity since the pandemic started to contain the advance of the transmission. It seems that testing individuals is even more critical since most countries are reopening their establishments, such as schools, restaurants, and shopping malls. The virus has been detected in various clinical specimen such as blood, crevicular fluid, urine, anal swabs ^2^ ^,^ ^3^. SARS-CoV-2 can infect multiple systems whose fluids can be used to test individuals for this disease. 

Even though nasopharyngeal swab (NPS) detects the presence of the virus itself, other forms of tracking COVID-19 have been tested and not achieving the same results ^2^ ^,^ ^4^. Therefore, the standard gold test for detecting SARS-CoV-2 is still the Real-Time - polymerase chain reaction (RT-PCR) performed from NPS. 

However, this test may be uncomfortable for the patient and requires specific training from the health professional responsible for collecting the sample and personal protective equipment. On the other hand, since a saliva specimen is a simple sample to collect, it would not require a trained professional. It could also be performed in children without significant complications because of it’s the self-collection way of obtaining the specimen. 

Thereby, saliva has been tested as an alternative way to collect samples that may be used to diagnose COVID-19 for both detection of infection, and for determining whether an infected individual is no longer capable of infecting and safe to encounter uninfected members of the community ^5^ ^;^ ^6^ ^;^ ^7^. To elucidate the versatility of non-invasive samples for testing for viral infections, studies with severe acute respiratory syndrome and Middle East respiratory syndrome coronavirus were performed ^7^. Therefore, saliva has been tested as an alternative for SARS-CoV-2 ^7^ ^,^ ^8^ ^,^ ^9^, especially in community settings ^5^. Thus, this study aimed to compare the results obtained from RT-PCR from NPS and saliva samples.

## Materials and methods

Three hundred and seventy-eight samples from Piquet Carneiro Polyclinic 189 outpatients were collected between September and November 2020. All of them had at least one symptom of Covid-19 at the time of collection and gave a written consent to participate. The Ethical Committee approved the study from Pedro Ernesto University Hospital (4.045.586). 

NPS were collected by trained health professionals and placed in conical tubes containing 3 mL of sterile saline solution. The saliva samples were collected by the patient him/herself in a sterile universal collector, using the spitting technique.

The 378 samples collected were sent and processed at the Histocompatibility and Cryopreservation Laboratory (HLA-UERJ). The materials were classified into two groups of 189 samples: NPS group and saliva group. The total nucleic acid was extracted from 200 µl of viral transport medium using the Bio-Gene DNA/ RNA Viral Extraction (Bioclin® Belo Horizonte, Minas Gerais, Brazil) isolation kit following the manufacturer's protocol.

The results were detected by the real-time reverse transcriptase-polymerase chain reaction (RT-PCR) technique with sequences of primers and probes from the molecular kit SARS-CoV-2 Bio-Manguinhos of the Berlin Protocol (Bio-Manguinhos, Rio de Janeiro, Brazil). The target-specific amplification methodology with fluorescence-labeled probes is used to determine the presence of the SARS-COV2 envelope gene and human RNAse P (RP) as an extraction control. The equipment used in the amplification and detection stage was the 7500 Real-Time PCR System (Applied Biosystems™ Waltham, Massachusetts, USA). The CT limit of detection was 42. 

The statistic software used in this study was SPSS (“Statistical Package for the Social Science”) version 23.0. Paired Student t-test was used to compare CT values from positive NPS and saliva samples. A Chi-square test was used to compare frequencies of symptoms in patients according to their NPS and saliva COVID-19 detection. The level of significance was set as 0.05.

## Results

There were 189 patients included in the present study (mean age: 39.6 ±12.3, 54 men and 143 female), with 378 samples (189 saliva and 189 NPS samples). Among the 189 swab samples, 142 had a non-detectable result (ND), while 47 had a detectable result (D). In addition, among the 142 "ND", 137 (94.4%) saliva samples obtained the same result, while 5 samples (3.4%) were “D”. Among the 47 “D” swab samples, 35 (74.4%) showed the same result as the saliva samples. The sensitivity value was 0.74 and the specificity was 0.97. The positive and negative predictive values were 0.88 and 0.92, respectively. The overall agreement between the NPS and saliva samples was 91% (172/189) (Table 1).

**Table 1:** SARS Covid-19 RT-PCR from nasopharyngeal (NPS) and saliva samples in symptomatic outpatients.

		NP swabs		Total
		Positive	Negative	
Saliva samples	Positive	35	5	40
	Negative	12	137	149
Total		47	142	189

A significantly higher frequency of patients referring loss of smell and taste (p=0.003), fever (p=0.003), and unwellness (p=0.02) was observed in positive samples as compared to negative NPS (Table 2). In saliva, a significantly higher frequency of patients referring fever (p=0.03) and unwellness (p=0.03) was observed in positive samples as compared to negative samples (Table 3). Among the 35 positive NPS and saliva samples (n=35), the mean (± SD) values of the cycle threshold (CT) of RT-PCR were 27.8 (± 4.3) and 30.6 (± 5.1), respectively (p=0.007).

**Table 2:** Mean (± SD) of age, gender distribution, and frequency of symptoms in outpatients with positive and negative nasopharyngeal samples (NPS) to detect COVID-19.

	Total (n=189)	NPS + (n=47)	NPS - (n=142)	p value
Age	39.2 (±11.5)	39.5 (±12)	38.1 (±9.8)	0.45
Gender female n (%)	141 (74.6)	34 (72.3)	107 (74)	0.7
Smoking yes n (%)	11 (5.8)	0 (0)	11 (7.7)	0.17
Unwellness yes n (%)	146 (77.2)	42 (89.4)	104 (73.2)	0.02
Cough yes n (%)	128 (67.7)	33 (70.2)	95 (67)	0.67
Loss of smell and/or taste n (%)	71 (37.5)	26 (55.3)	45 (31.7)	0.003
Abdominal pain yes n (%)	70 (37)	18 (38.3)	52 (36.6)	0.83
Fever yes n (%)	86 (45.5)	30 (63.8)	56 (39.4)	0.003

NPS +: individuals who tested positive on the NPS test;

NPS -: individuals who tested negative on the NPS test.

p-value: differences among positive and negative samples

**Table 3:** Mean (± SD) of age, gender distribution, and frequency of symptoms in outpatients with positive and negative saliva samples to detect COVID-19.

	Total (n=189)	saliva + (n=40)	Saliva - (n=149)	p value
Age	39.2 (±11.5)	38.3 (±10.2)	39.4 (±11.8)	0.6
Gender Female n (%)	139 (73.5)	30 (75)	109 (73.2)	0.8
Smoking n (%)	11 (5.8)	0 (0)	11 (7.4)	0.26
Unwellness n (%)	149 (78.8)	36 (90)	110 (73.8)	0.03
Cough n (%)	128 (67.7)	26 (65)	102 (68.5)	0.67
Loss of smell and/or taste n (%)	71 (37.6)	20 (50)	51 (34.2)	0.06
Abdominal pain n (%)	70 (37)	15 (37.5)	55 (36.9)	0.95
Fever n (%)	86 (45.5)	24 (60)	62 (41.6)	0.03

Saliva +: individuals who tested positive on the saliva test.

Saliva -: individuals who tested negative on the saliva test.

p-value: differences among positive and negative samples

## Discussion

This study aimed to compare the results obtained from NPS and saliva samples using RT-PCR. The results showed that the sensitivity value was 0.74, and the specificity was 0.97. The positive and negative predictive values were 0.88 and 0.92, respectively. Two studies specifically compared both samples ^10^ ^,^ ^11^. Jamal et al. ^10^ examined 91 inpatients with COVID-19 and tested one pair of NPS/saliva samples from each patient using RT-PCR. They reported that sensitivity was 0.89 for NPS samples and 0.72 for saliva samples. However, the authors did not describe specificity and positive and negative predictive values. If we calculate positive and negative predictive values, they were 0.84 and 0.48. Differences may be related to the different periods that the samples were collected after the onset of the illness. Landry et al. ^11^ tested paired samples of NPS/saliva, in a cross-sectional study including 124 symptomatic outpatients. Out of these 124, 28 were positive for both saliva and NPS samples. Two patients were negative for NPS samples and positive for saliva samples, while 5 were positive just for the NPS sample. The overall agreement between NPS and saliva samples was 94.4%, similar to the agreement of the present study (91%). The sensitivity for saliva was 0.85. Both studies did not report positive and negative predictive values. After a laboratory test, these results would be interesting if one wants to identify patients with a disease.

To et al. ^9^ examined 12 patients hospitalized with laboratory-confirmed SARS-CoV-2 and informed that 11 saliva samples were also positive to detect the virus. However, their sample size was small. They aimed to verify the presence of the virus in the saliva, not to compare NPS and saliva samples.

Wyllie et al. ^12^ aimed to validate the use of saliva for SARS-CoV-2 detection. The authors found that the sensitivity of saliva SARS-CoV-2 detection is superior to nasopharyngeal swabs in early hospitalization and more consistent during extended hospitalization and recovery. More recently, Sakanashi et al. ^13^ used 12-paired NPS and saliva specimens collected from patients at various time points after symptom onset. Their results suggested that saliva samples were a practical non-invasive alternative to NPS in detecting SARS-CoV-2 using RT-PCR.

Another study tested pooled saliva in 449 individuals, using the same CT values from RT-PCR. On the other hand, they have found that saliva testing is not quite as sensitive as nasopharyngeal and midturbinate testing ^14^. They still highlighted that higher viral load in an asymptomatic screening program is adequate to detect saliva.

CT values were significantly lower in NPS samples than saliva samples, similar to what was described by Williams et al and Landry et al. ^11^. Saliva is a biological fluid that may be used as a non-invasive diagnostic technique. It may be self-collected, does not cause patient discomfort, protects the health professionals, and reduces the cost of having a specific team for collecting and using protective equipment. Azzi et al. ^15^ examined 25 saliva samples of inpatients with severe or very severe COVID-19. The authors suggested that saliva might be a promising tool in COVID-19 diagnoses.

Other forms of analyzing the SARS-CoV-2 have been suggested. Reverse transcription loop-mediated isothermal amplification, which is a gene amplification procedure, has been tested. Compared to RT-PCR, it was demonstrated to be a simple, rapid, specific, and cost-effective nucleic acid amplification method ^16^ ^,^ ^17^. However, RT-PCR is routinely used to detect causative viruses from respiratory secretions due to its laboratory diagnostic reliability ^18^. 

In conclusion, detection of Sars-CoV-2 using saliva samples showed high sensitivity and specificity compared with nasopharyngeal swabs.
